# Changes in daily routines and health-related quality of life among Spanish older adults after the COVID-19 pandemic: analysis from a gender perspective

**DOI:** 10.3389/fpubh.2025.1678003

**Published:** 2025-10-13

**Authors:** Candela Cameselle-Lago, Milagros Rico-Blázquez, Raquel Sánchez-Ruano, Alba Ayala, Marcos Pascual-García, María Solé-Agustí, Iraida Gimeno-Pi, Aitziber Echevarria-Echevarria, Víctor M. González-Chordá, Silvia Esteban-Sepúlveda, Azucena Pedraz-Marcos, María Consuelo Company-Sancho, María Ángeles Cidoncha-Moreno, María Clara Vidal-Thomas, Joana Maria Taltavull-Aparicio, María Teresa Moreno-Casbas, CUIDAMOS+75 Group, Almudena Avendaño Céspedes, Elisa Belén Cortés Zamora, Gemma Hidalgo Alsina, Marta Puig Fitó, Maria Rosa Rami Badia, Maria Eugènia Obis Aguilar, Anna Guiu Bueria, Maria Teresa Palau Morales, Inmaculada Fabregat Julvé, Clara Bernabeu Aicart, Juan Miguel Mestre Tena, Marta Tomas Brea, María Dolores Parreño Membrado, Carla Gimeno Escuder, Ana Moliner Monedero, Alberto Cano Pérez, Anunciación Martínez Arroyo, Carmen Luzdivina Sánchez Ramos, Carolina Regacho Ramírez, Consuelo Carretero Sierra, Cristina Villanueva Sanz, David Verdagay Romero, Diego Ruiz García, Elena Monteagudo García, Elena Rojas Barra, Elvira Herrero Sancho, Esther Luna Espejo, Gema Díez Rodríguez, Gloria Leyva Vera, Gonzalo Nuevo Pozo, Ines Melero Redondo, Inmaculada Moreno García-Herrera, Isabel Careaga González, Laura Villanova Cuadra, Lorena Ríos Ruiz, Luis Huerta Galindo, María Ángeles Almodovar López, María Concepción Hernández de Luna, María Isabel Adillo Montero, María Jesús Santamaría Martín, María Luisa Romero Molina, Nadia Kamal Moreno, Paloma Garrido Calleja, Pedro Vadillo Obesso, Raquel López Zarza, Raquel Razola Rincón, Rocío López Sánchez, Samuel Lobato Márquez, Sara Criado Jorge, Silvia Morcillo de la Cuadra, Teresa Susmozas Rodríguez Barbero, Ana María Carpio Ibáñez, Elena García Gómez de Cadiñanos, Maria Consuelo Company Sancho, Rita Mendoza Sánchez, María José García Delgado, Guillermo Monzón Monzón, Maria Isabel Díaz Díaz, María Dolores Suárez Hernández, Beatriz Candela Angulo, Maria Paula Martín Andrade, Pedro Jorge Araujo, Eduardo Santana Aguiar, Sandra Morales Montesdeoca, Laura Figueroa Martín, Haridian Galdona Luis, Silvia Martínez Mata, Rosa Mellado Tirado, Irene Parrilla Suárez, Sara Ramos Santana, Victoria Plasencia Delgado, Ingrid Rodríguez Hernández, Jorge Rafael Padilla Maestre, Sergio Garrido Bollo, Teresa Martínez de la Torre, Inmaculada Guzmán Castilla, María Artieda Cadena, Vanesa Ayesa Urtiaga, Itziar Berasain Erro, Anamaría Zubillaga Beorlegui, Graciela Ilundain Razquin, María Carmen Gutiérrez Pardo, Maite Lucea Matinez, Mónica Magno Villanueva, María de Leyre Martínez Zaborras, María Jesús Martínez Cabodevilla, María José Galán Espinilla, Ana María Ariztegui Echenique, Ana María Mateo Cervera, Mª Ángeles Cidoncha Moreno, Maribel Moreno Leal, Ana Isabel Barrenetxea Lejarreta, Asier Albestegi Soto, María Félix Iglesias Domínguez, Ruth Gallo Alcalá, Elisabet Carrera Alfonso, Mara Pisà Gaia, Jerónima Miralles Xamena, Milagros Sánchez Jiménez, M. Josep Barea Mestre, Elena García Salom, Nuria Garcia Duran, Rosa María Pons Burguera, Wiliam Andres Severi, Tamara Góméz Bermejo, Ana Belén Campos Guerrero, Marta Perelló Fuster, M. Soledad Hernandez Yeste, Elena Terrón Rodriguez, Laura De la Rosa López, Encarnación Cantos Garcia, Fuensanta Arjona Hernández, Maria Milagros De Castro Día, Pilar Polo Quiles, Rita Isabel Soriano Campos, Josefa Chumillas López, Maria Jose Fernandez Abellan, Maria Rosario Valverde Jimenez, Maria Jose Muñoz Garcia, Margarita Cerezo Sanmartin, Dolores Morales Nicolas, Maria Jose Laveda Lopez

**Affiliations:** ^1^Nursing and Healthcare Research Unit (Investén-isciii), Instituto de Salud Carlos III, Madrid, Spain; ^2^Escuela Internacional de Doctorado de la Universidad Nacional de Educación a Distancia (UNED), Programa de Doctorado en Ciencias Biomédicas y Salud Pública, Madrid, Spain; ^3^Research Unit, Primary Care Assistance Management, Madrid Health Service, Madrid, Spain; ^4^Research Network on Chronicity, Primary Care and Health Promotion -RICAPPS-(RICORS), ISCIII, Madrid, Spain; ^5^Gregorio Marañon Health Research Institute, Madrid Health Service, Madrid, Spain; ^6^UCM Research Group ‘Public Health-Lifestyles, Nursing Methodology, and Care in the Community Environment’, Nursing Department, Faculty of Nursing, Physiotherapy and Podiatry, Complutense University of Madrid, Madrid, Spain; ^7^Foundation for Biosanitary Research and Innovation in Primary Care of the Community of Madrid (FIIBAP), Madrid, Spain; ^8^University Institute on Gender Studies, University Carlos III of Madrid, Madrid, Spain; ^9^Alpes Healthcare Centre, Primary Care Assistance Management, Madrid Health Service, Madrid, Spain; ^10^Subdirección continuidad de cuidados Área I, Servicio Murciano de Salud, Murcia, Spain; ^11^Instituto Murciano de Investigación Biosanitaria Pascual Parrilla (IMIB), Murcia, Spain; ^12^Gerència d'Atenció Primària i a la Comunitat Lleida (GAPiC), Catalan Health Institute (ICS), Barcelona, Spain; ^13^Institut d'Investigació en Atenció Primària de Salut Jordi Gol, Barcelona, Spain; ^14^Patient Care Unit, OSI Barrualde Galdakao, Gadalkao, Spain; ^15^Grupo de Investigación en Enfermería (GIENF-281), Universitat Jaume I, Castelló, Spain; ^16^Centro de Investigación Biomédica en Red (CIBERFES), Madrid, Spain; ^17^Departament d’Infermeria Fonamental i Clínica, Facultat d’Infermeria, Universitat de Barcelona (UB), L’Hospitalet de Llobregat, Barcelona, Spain; ^18^Dirección General de Salud Pública, Servicio Canario de la Salud, Las Palmas, Spain; ^19^IIS Biobizkaia, General Head Office of Osakidetza, Vitoria-Gasteiz, Spain; ^20^Primary Care Research Unit of Mallorca (IB-Salut), Palma de Mallorca, Spain

**Keywords:** COVID-19, older adults, daily routines, health-related quality of life, gender perspective, EQ-5D-5L, primary care

## Abstract

**Aims:**

To examine the association between perceived changes in daily routines due to the COVID-19 pandemic and health-related quality of life (HRQoL) among community-dwelling adults aged 75 and older in Spain, through a gender-stratified analysis.

**Design:**

Cross-sectional, community-based, multicentre study using baseline data from the CUIDAMOS+75 cohort.

**Methods:**

A total of 1,072 older adults over 75 from 11 Spanish regions were interviewed between June 2022 and June 2023. Data on SARS-CoV-2 exposure, sociodemographic and clinical characteristics and perceived changes in daily routines (basic-needs, healthcare and social routines) were collected. HRQoL was measured using the EQ-5D-5L utility index. Gender-stratified linear regression models were used to examine associations between changes in routines and HRQoL.

**Results:**

Over half of the participants (52%) reported considerable changes in their daily routines due to the pandemic, especially in social routines (48%). Women reported more frequent changes across all routine types. Although women had lower overall HRQoL scores, changes in routines were more strongly associated with poorer HRQoL among men, particularly when healthcare routines were affected. Among women, significant associations were observed for disruptions in combined basic and social routines as well as when all three routine types were simultaneously affected. Notably, COVID-19 infection status was not associated with HRQoL after adjustment.

**Conclusion:**

The COVID-19 pandemic led to substantial and persistent changes in daily routines among older adults in Spain, with gender-specific patterns in their association with HRQoL. These findings highlight the importance of incorporating a gender perspective in public health responses to health crises, particularly in strategies aimed at preserving daily routines to support autonomy and wellbeing in older populations.

## Introduction

The COVID-19 pandemic presented an unprecedented challenge to societies around the world. In response, authorities implemented drastic public health measures to contain the spread of the virus, including particularly severe restrictions in countries such as Spain, where citizen mobility was limited and home confinement was mandated. A state of alarm was declared in Spain from 14 March to 21 June 2020, with sequential phases for lifting mobility restrictions, adjusted by region based on epidemiological data (1). The virus containment measures, especially home confinement, proved to be effective (2), although they also resulted in considerable social impact (3) and detrimental effects on physical activity, sleep quality and mental health and wellbeing (4). These measures, combined with the unpredictable course of the pandemic, triggered and exacerbated natural responses such as fear, loneliness, stress and anxiety, particularly among vulnerable groups, including children, migrants, individuals at risk of exclusion, people with psychiatric conditions, older adults and caregivers (5).

The global outbreak of COVID-19 posed distinct challenges for older adults. On the one hand, advanced age was associated with higher mortality rates from SARS-Cov-2 infection, contributing to heightened fear of contagion within this population group (5). On the other hand, containment measures led to a substantial reduction in traditional forms of social interaction with relatives, replaced by increased use of digital tools for communication. In this context, older adults experienced a marked decline in social engagement—partly due to fear of face-to-face interactions, and partly due to the barriers many faced in accessing and using digital communication tools (6). Additionally, individuals with multiple chronic conditions or pre-existing psychiatric disorders encountered further difficulties in accessing health and social care services, due to both the burden on the healthcare system during the health emergency and the deterioration of informal community support networks (7).

Additionally, older adults experienced substantial changes in their daily routines, which affected key dimensions of their quality of life, including mobility, autonomy and social interactions. Everyday activities such as grocery shopping, attending personal care appointments or managing errands were suspended or significantly altered due to the containment measures. In Spain, social gatherings and outdoor activities were also restricted during periods of high viral incidence, leading to reduced opportunities for group-based or outdoor physical activity, as well as limiting interpersonal contact (8). Moreover, access to essential healthcare services—such as medical consultations, rehabilitation or support for chronic conditions—was frequently disrupted. Many older adults, especially women, faced difficulties in providing informal care to others, such as dependent spouses or grandchildren, due to the fear of contagion and mobility restrictions (9).

The disruption of daily activities during the pandemic led to a marked decline in social participation among older adults, which has been linked to a deterioration in both emotional and cognitive health (10). In this population, maintaining daily routines and social participation are crucial drivers of physical activity, as they promote light physical exercise and help prevent sedentary behaviours (9). Moreover, preserving daily routines enhances autonomy and fosters a sense of self-sufficiency and self-determination, contributing to a greater life satisfaction and overall quality of life (11).

Health-related quality of life (HRQoL) is a multidimensional health indicator which considers individuals’ perception of their health status as well as their position in life. It encompasses physical health, psychological wellbeing, functional independence, social relationships and the relationship with the environment. HRQoL is influenced by a wide range of factors, including functional status and physical activity, psychological health, perceived social support and levels of social participation (12).

Given its multidimensional nature, several studies have assessed the impact of the COVID-19 pandemic on HRQoL. A systematic review analysing HRQoL before and during the COVID-19 pandemic concluded that the pandemic had a negative impact on quality of life across the worldwide general population (13). In a study conducted with community-dwelling older adults in Japan, reductions in physical activity due to the pandemic were associated with significantly lower quality of life scores, confirming the relevance of maintaining physical activity in this age group, even in the context of a public health emergency (14). Similarly, a study involving patients with multiple sclerosis found that those who had discontinued a greater number of daily activities due to the pandemic reported lower HRQoL scores than those who had maintained their routines (15).

Furthermore, HRQoL is strongly influenced by sociodemographic factors such as gender, with women generally reporting lower HRQoL scores across all age groups (16). These differences have been partially explained by social factors related to gender roles, as well as by potentially modifiable factors such as body mass index (BMI) and lifestyle determinants, including physical activity (17). Previous studies have shown that men and women have adjusted their activities differently in response to the COVID-19 pandemic. In this regard, women have demonstrated greater adaptability and compliance with public health measures than men, leading to a larger reduction in daily activities (18).

Based on this background, we hypothesised that women and men in Spain may have experienced different patterns of change in their daily routines because of the COVID-19 pandemic. We also hypothesised that changes or interruptions in the daily routines of older adults in Spain could be associated with a poorer health-related quality of life in this age group.

The aim of this study was to examine, in a cohort of individuals aged 75 years and older receiving primary healthcare across 11 Spanish regions, the association between changes in basic, healthcare-related, and social routines due to the COVID-19 pandemic and self-perceived quality of life, as measured with the EQ-5D-5L utility index, through a gender-stratified analysis. Secondary aims included describing the frequency and patterns of changes in routines, as well as identifying the sociodemographic and clinical determinants associated with these changes.

## Methods

### Study design and setting

This cross-sectional, community-based, multicentre study is based on baseline data from the CUIDAMOS+75 cohort, a mixed-methods project designed to assess the impact of the COVID-19 pandemic on individuals aged 75 years and older in Spain. This analysis focuses on the associations between perceived changes in daily routines and Health-Related Quality of Life (HRQoL). Full methodological details are available in the published protocol (19). The STROBE checklist was followed in the preparation of this manuscript and is provided as supporting information (Additional file 1).

### Participants

Eligible participants were non-institutionalised individuals aged 75 years and older with an active electronic health record in the Spanish National Health System, residing in 11 autonomous regions (Andalusia, Balearic Islands, Canary Islands, Cantabria, Castilla-La Mancha, Catalonia, Madrid, Valencia, Murcia, Navarre, and the Basque Country). Inclusion required the ability to complete the interview and provide written informed consent. Exclusion criteria included moderate to severe cognitive impairment (confirmed through clinical history or a Pfeiffer score ≥ 5) (20), severe sensory deficits, documented severe psychiatric illness, a life expectancy of ≤ 9 months (i.e., included in palliative care), or total functional dependence (Barthel Index ≤ 5 points).

Participants were consecutively invited to participate by their usual nurses during routine primary care visits or home assessments. Recruitment took place between June 2022 and June 2023.

### Sample and sampling procedure

The estimated sample size was 1,035 participants, based on the projected availability of eligible individuals, and assuming a 2:1 ratio of non-exposed to exposed individuals to SARS-CoV-2. Recruitment continued until the planned sample size was reached.

Participants were classified as exposed if they had a confirmed SARS-CoV-2 infection (positive rRT-PCR or antigen test performed within 5 days of symptom onset) between 11 May 2020 and 1 June 2022. Those with no recorded infection during this period were considered as non-exposed. This date was chosen because it coincided with the start of participant recruitment and reflected a notable change in the epidemiological situation in Spain, including a decrease in COVID-19 incidence following the Omicron BA.4/BA.5 wave and the end of mandatory quarantines for most of the population. Individuals who experienced infection after this date were therefore classified as “non-exposed.” Further details on sample assumptions are available in the study protocol (19).

### Data collection procedure and variables

Data were collected through structured interviews conducted by trained nurses and recorded in an electronic data system between June 2022 and June 2023. The primary outcome was HRQoL, assessed using the EQ-5D-5L instrument. This standardised tool includes a descriptive system comprising five dimensions (mobility, self-care, usual activities, pain/discomfort, and anxiety/depression) each with five levels of severity, as well as a visual analogue scale (EQ-VAS) ranging from 0 to 100. A weighted utility index was calculated for each participant using the value set for the Spanish population (16). The questionnaire’s descriptive system allows the definition of 3,125 different health profiles, ranging from the best health status “11,111” to the worst “55,555.”

Variables were grouped into sociodemographic, clinical, pandemic-related or routine-related and HRQoL. Sociodemographic variables included age at inclusion, self-identified gender (woman, man or non-binary), and cohabitation status (living alone or with others).

Clinical information included the number of chronic conditions (categorised by body system), body mass index at the time of the interview, presence of a regular caregiver (formal or informal, cohabiting or not), and perceived social support measured with the Duke-UNC questionnaire (21).

The COVID-19 exposure variable was confirmed SARS-CoV-2 infection by rRT-PCR or antigen testing with recent symptoms. Additional pandemic-related variables included the loss of a family member or close friend due to COVID-19 (Yes/No), and threat perception assessed using the BIP-Q5 questionnaire (range 0–50) (22). Functional status was measured using the Barthel Index (23), and anxiety symptoms were assessed with the Hamilton Anxiety Scale (24).

The questions used to assess the impact of the COVID-19 pandemic on daily routines among older adults were specifically developed for this study, based on the expertise of clinical professionals and researchers in geriatrics, primary care, and public health. They consisted of three questions regarding basic, healthcare-related, and social routines. Responses were reclassified into “no or little change” versus “moderate or considerable change,” and a composite variable with eight possible combinations was constructed.

Health-related quality of life was assessed with the EQ-5D-5L, including both the EQ-VAS score and the utility index. A complete list of the questions and response categories used during the interviews is provided in Additional file 2.

### Statistical analysis

Data were analysed to describe the sociodemographic, clinical, and COVID-19-related characteristics of the study population according to gender and COVID-19 exposure status. Descriptive analysis was performed using means and standard deviations for continuous variables, and frequencies and percentages for categorical variables. Differences between groups were assessed using the Wilcoxon rank-sum test for continuous variables and the chi-square test for categorical variables, with a significance level of 5%. The sociodemographic and clinical factors associated with changes in routines were assessed. For each of the three types of routines (related to basic needs, healthcare, and social routines), participant characteristics were compared according to whether they reported changes. Adjusted odds ratios (aOR) were estimated using logistic regression models, controlling for age and gender.

Separate linear regression models were constructed for men and women to examine the association between changes in routines and HRQoL. Covariates were selected based on their statistical association in the bivariate analysis and theoretical relevance, retaining those that remained significant in the multivariate models, together with the main exposure variable of interest (SARS-CoV-2 infection status). The dependent variable was the EQ-5D-5L utility index, and independent variables included SARS-CoV-2 exposure status, age, change in routines (composite variable), number of chronic conditions, Barthel Index, Hamilton Anxiety Rating Scale, BMI and region of residence. Geographic heterogeneity was accounted for by including region (11 autonomous regions) fixed effects. The assumptions of the linear regression model were assessed and met. Statistical analyses were conducted using RStudio 2023.12.1. All variables were standardised prior to analysis to generate standardised B coefficients, in order to facilitate the interpretation and comparison of coefficients obtained in multivariate regression.

### Ethical considerations

All participants provided written informed consent. The study was conducted in accordance with the fundamental ethical principles of autonomy, beneficence, justice and non-maleficence, and adhered to Good Clinical Practice standards, the Declaration of Helsinki (2024) and the Oviedo Convention (1997). The processing, communication and transfer of data were carried out in compliance with current data protection regulations in force (25) and the rights of data access, rectification, cancellation, and opposition (ARCO) contemplated in that law.

The coordinated study received approval from the Central Research Committee of Primary Healthcare Management (June 2022) and the Ethics Committee for Drugs Research (CEIm) of the University Hospital La Princesa in June 2022. All participating regions obtained approval from their respective ethics committees.

## Results

### Participant characteristics

A total of 1,072 community-dwelling adults aged 75 and older were included in the final analytical sample, after excluding 10 individuals whose reported SARS-CoV-2 infection dates fell outside the eligibility window. A total of 1,029 participants were included in the final analysis. We verified that 3.2% of participants had missing values in at least one of the variables included in the final model.

The sample was predominantly female (59.2%), with a median age of 82 years (range 75–101). Two participants identified as non-binary; they were classified as missing for the gender variable and therefore excluded from stratified and regression analyses where gender was included as a covariate. However, they were retained in the overall descriptive statistics of the study sample. Most participants lived with other individuals (72%), although women were significantly more likely to live alone than men (37% vs. 16%, *p* < 0.001). Women also had a higher mean number of chronic conditions (2.9 vs. 2.7, *p* = 0.026) and were more likely to report having a regular caregiver (42% vs. 27%, *p* < 0.001).

Regarding COVID-19 exposure, 297 (27.7%) participants had a confirmed infection. Exposed individuals were more likely to live with others (77% vs. 70%, *p* = 0.028) and to report the loss of a close relative due to the pandemic (27% vs. 20%, *p* = 0.014).

Perceived threat from COVID-19 was slightly higher among women (mean BIP-Q5 score: 24.0 vs. 23.0, *p* = 0.030), but no significant differences were observed in functional status (Barthel Index), social support (Duke-UNC), or anxiety levels (Hamilton Anxiety Rating Scale) between exposure groups. However, women showed significantly higher anxiety scores (8.0 vs. 5.0, *p* < 0.001) and a greater prevalence of moderate or severe dependence in activities of daily living (*p* = 0.014).

Health-related quality of life, measured by the EQ-5D-5L index, was significantly lower in women compared to men (0.8 vs. 0.87, *p* < 0.001), and slightly lower in the exposed group compared to the non-exposed group (0.77 vs. 0.81, *p* = 0.017).

Sociodemographic, clinical, and pandemic-related variables of the participants, disaggregated by gender and exposure status, are presented in Table 1.

**Table 1:** Sample description.

Characteristic	By exposure to COVID-19				By gender		
	Overall, *N* = 1,072^a^	Exposed, *N* = 297^a^	Non-exposed, *N* = 775^a^	*p*-value^b^^,^^c^	Men, *N* = 428^a^	Women, *N* = 635^a^	*p*-value^b^
Age (years)	82.7 (4.4)	82.8 (4.3)	82.6 (4.4)	0.500	82.3 (4.2)	82.9 (4.5)	0.066
Home cohabitation status				0.028			<0.001
With other/s	764 (72%)	227 (77%)	537 (70%)		359 (84%)	398 (63%)	
Alone	300 (28%)	69 (23%)	231 (30%)		68 (16%)	230 (37%)	
Number of conditions (by body system)	2.8 (1.63)	3.0 (1.68)	2.7 (1.61)	0.075	2.9 (1.67)	2.7 (1.59)	0.026
BMI	27.9 (4.7)	28.1 (4.7)	27.9 (4.7)	0.500	27.7 (4.5)	28.1 (4.8)	0.090
Has a caregiver	378 (35%)	121 (41%)	257 (33%)	0.019	114 (27%)	263 (42%)	<0.001
Lost a relative to the pandemic	230 (22%)	78 (27%)	152 (20%)	0.014	98 (23%)	129 (20%)	0.300
COVID-19 threat perception (BIP-Q5)	24.0 (11)	23.0 (11)	24.0 (11)	0.062	23.0 (11)	24.0 (11)	0.030
Changes in routines related to basic needs				0.130			<0.001
Little or no change	789 (74%)	209 (71%)	580 (75%)		344 (80%)	441 (70%)	
Considerable or substantial change	279 (26%)	87 (29%)	192 (25%)		84 (20%)	190 (30%)	
Changes in healthcare routines (for self or others)							<0.001
Little or no change	718 (67%)	190 (64%)	528 (68%)		318 (74%)	395 (63%)	
Considerable or substantial change	350 (33%)	106 (36%)	244 (32%)		110 (26%)	236 (37%)	
Changes social engagement routines				0.900			0.055
Little or no change	556 (52%)	155 (53%)	401 (52%)		239 (56%)	314 (50%)	
Considerable or substantial change	511 (48%)	140 (47%)	371 (48%)		189 (44%)	316 (50%)	
ADL independence (Barthel Index)				0.200			0.014
Independent	1,018 (96%)	277 (95%)	741 (96%)		416 (98%)	593 (94%)	
Moderate dependence	31 (2.9%)	9 (3.1%)	22 (2.9%)		6 (1.4%)	25 (4.0%)	
Severe dependence	15 (1.4%)	7 (2.4%)	8 (1.0%)		3 (0.7%)	12 (1.9%)	
Social support (Duke-UNC)	46.0 (8)	46.0 (9)	46.0 (8)	0.900	46.0 (8)	46.0 (9)	0.500
Hamilton anxiety rating scale	6.0 (8)	7.0 (8)	6.0 (7)	0.200	5.0 (6)	8.0 (8)	<0.001
EQ-5D-5L utility index	0.80 (0.21)	0.77 (0.22)	0.81 (0.20)	0.017	0.87 (0.15)	0.75 (0.22)	<0.001

a Mean (SD); *n* (%).

b Wilcoxon rank sum test; Pearson’s Chi-squared test.

c Fisher’s exact test.

### Changes in daily routines and associated factors

More than half of the participants (56.1%) reported experiencing moderate or substantial changes in at least one type of daily routine due to the COVID-19 pandemic. Social routines were the most frequently affected (48%), followed by healthcare-related (33%), and basic needs routines (26%). Furthermore, 18% of participants reported disruptions across all three domains simultaneously.

Gender differences were notable. Women were significantly more likely than men to report changes in basic routines (30% vs. 20%, *p* < 0.001) and healthcare-related routines (37% vs. 26%, *p* < 0.001). A higher frequency of change in social routines was also observed among women (50% vs. 44%), although this difference was not statistically significant.

Several factors were associated with a higher likelihood of reporting moderate or substantial changes in routines (Table 2). Participants who had lost a close relative due to COVID-19 were significantly more likely to report disruptions across all routine types (basic: 33% vs. 24%; healthcare: 45% vs. 29%; social: 62% vs. 44%; all *p* < 0.01). Similarly, higher perceived threat from COVID-19, as measured by the BIP-Q5, was consistently associated with greater routine disruption (*p* < 0.001 across all domains).

**Table 2:** Sociodemographic and clinical factors associated with changes in routines.

Characteristic	Change in routines related to basic needs		Changes in healthcare routines		Changes in social routines	
	Frequency^a^ or mean difference^b^	Adj. OR^c^ (confidence interval)	Frequency^a^ or mean difference^b^	Adj. OR^c^ (confidence interval)	Frequency^a^ or mean difference^b^	Adj. OR^c^ (confidence interval)
Age (years)	82.3 vs. 82.8	**0.968 (0.936–0.999)**	82.6 vs. 82.7	0.993 (0.964–1.020)	82.6 vs. 82.7	0.992 (0.965–1.020)
Gender						
Men = 428	84 (20%)		110 (26%)		189 (44%)	
Women = 635	**190 (30%)**	**1.795 (1.340–2.420)**	**236 (37%)**	**1.731 (1.320–2.280)**	316 (50%)	1.280 (0.999–1.640)
Cohabitation status						
With other/s = 764	200 (26%)		242 (32%)		364 (48%)	
Alone = 300	79 (26%)	0.891 (0.647–1.220)	108 (36%)	1.050 (0.785–1.410)	144 (48%)	0.985 (0.747–1.300)
Social support (Duke-UNC)	**44 vs. 47**	**0.958 (0.943–0.973)**	**45 vs. 47**	**0.965 (0.950–0.980)**	**45 vs. 48**	**0.963 (0.949–0.977)**
COVID-19 exposure						
Non-exposed = 309	192 (25%)		244 (32%)		371 (48%)	
Exposed = 763	87 (29%)	1.250 (0.920–1.690)	106 (36%)	1.210 (0.908–1.610)	140 (47%)	0.965 (0.736–1.260)
Time since interview (months)	23.8 vs. 23.9	0.988 (0.947–1.030)	24.0 vs. 23.8	1.030 (0.989–1.070)	23.8 vs. 24.0	0.980 (0.944–1.020)
Lost a relative to the pandemic						
No = 835	202 (24%)		245 (29%)		366 (44%)	
Yes = 230	**75 (33%)**	**1.580 (1.140–2.179)**	**103 (45%)**	**2.092 (1.540–2.840)**	**143 (62%)**	**2.132 (1.580–2.890)**
COVID-19 threat perception (BIP-Q5)	**27 vs. 23**	**1.031 (1.020–1.040)**	**26 vs. 23**	**1.029 (1.020–1.040)**	**25 vs. 22**	**1.024 (1.010–1.040)**
Number of conditions	2.91 vs. 2.75	1.090 (0.999–1.190)	2.89 vs. 2.74	1.080 (0.995–1.170)	2.84 vs. 2.75	1.04 (0.963–1.120)
BMI	27.8 vs. 28.0	0.988 (0.959–1.020)	27.8 vs. 28.0	0.987 (0.960–1.01)	27.8 vs. 28.0	0.988 (0.962–1.010)
ADL Independence (BI)	**90 vs. 93**	**0.984 (0.974–0.994)**	**90 vs. 93**	**0.988 (0.979–0.998)**	92 vs. 93	0.993 (0.984–1.000)
Has a caregiver						
No = 692	**156 (23%)**		**194 (28%)**		323 (47%)	
Yes = 378	**123 (33%)**	**1.907 (1.400–2.600)**	**156 (41%)**	**1.889 (1.410–2.530)**	188 (50%)	1.160 (0.885–1.530)

a *n*(%) of participants who reported a considerable or substantial change in each category.

b Mean comparison between the participants who reported a considerable or substantial change compared to (vs.) those who did not.

c Adjusted for age and gender. Bold values indicate statistical significance (*p* < 0.05).

Lower perceived social support was also associated with changes in routines. Participants reporting changes in basic, healthcare, or social routines had significantly lower Duke-UNC scores compared to those without changes (mean differences of 2–3 points, all *p* < 0.001).

Functional status was another key factor. Individuals with lower Barthel Index scores were more likely to report changes in basic and healthcare routines (mean score: 90 vs. 93, *p* < 0.01). Additionally, having a caregiver was associated with a higher likelihood of reporting changes in both basic (33% vs. 23%, *p* < 0.001) and healthcare routines (41% vs. 28%, *p* < 0.001).

No significant associations were found between changes in routines and age (except a weak inverse association with basic routines after adjusting for gender), cohabitation status, number of chronic conditions, BMI, or COVID-19 infection status.

### Gender differences in HRQoL and their relationship with changes in routines

Of the 1,072 individuals included in the study, 1,065 (426 men and 630 women) completed all items of the EQ-5D-5L questionnaire, allowing for the calculation of utility scores. The dimension with the highest prevalence of reported problems was pain/discomfort, affecting 65% of the total sample (53% of men vs. 73% of women, *p* < 0.05). Mobility (35% in men vs. 50% in women, *p* < 0.01) and anxiety/depression (22% vs. 47%, *p* < 0.05) were the next most frequently affected dimensions. Participants aged 85 and older reported a higher prevalence of problems across all dimensions except pain. The lowest EQ-5D-5L index scores were observed among women aged ≥85 (0.69), followed by women <85 (0.78), men ≥85 (0.83), and men <85 (0.88).

**Figure 1:**
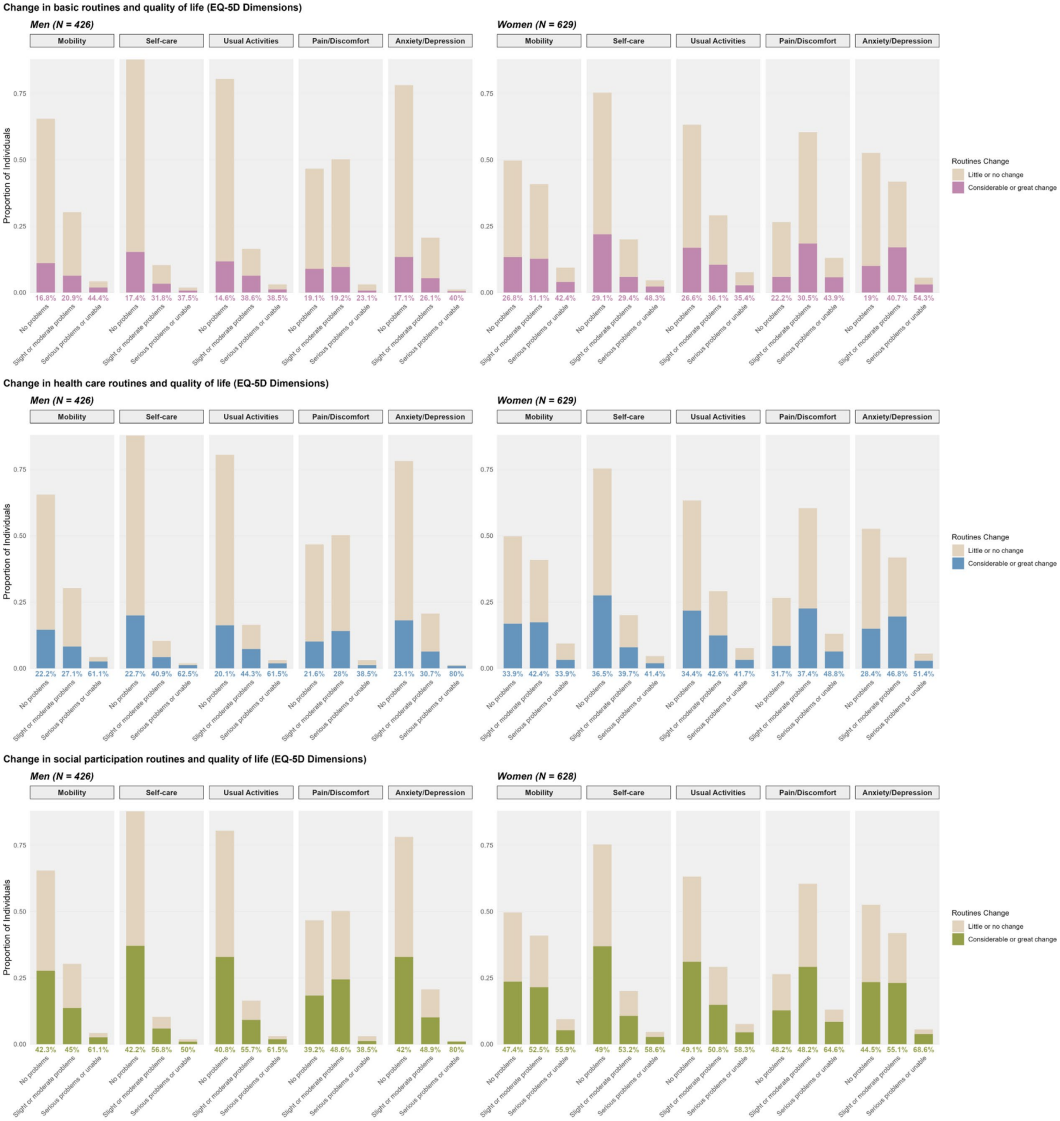
Changes in routines and EQ-5D-5L dimensions.

Figure 1 displays the distribution of response across EQ-5D-5L dimensions disaggregated by gender along the proportion of participants reporting changes in daily routines (basic, healthcare and social), categorised as little or no change versus moderate or substantial change. Women showed a higher prevalence of problems across all EQ-5D dimensions, with the largest gender gap observed for Anxiety/depression.

In general, participants who reported problems in any EQ-5D dimension were more likely to report changes in their routines. Among women, this pattern was less pronounced. Even those without reported problems in EQ-5D dimensions showed a relatively high prevalence of changes in routines. However, stronger associations were observed between changes in routines and problems in the dimensions of pain/discomfort and anxiety/depression. Specifically, women experiencing greater levels of pain or more severe anxiety/depression were more likely to report changes in their routines. In contrast, among men, reporting no problems in Mobility, Self-care, or Usual Activities was associated with a greater tendency to report little or no change in basic and healthcare routines.

Adjusted linear regression results for the EQ-5D-5L utility index, stratified by gender, are presented in Table 3. Although COVID-19 exposure status was statistically significant in the bivariate analysis, it was not associated with HRQoL after adjustment in either model. In the model for men, older age (B* = −0.137, *p* < 0.001), the number of chronic conditions (B* = −0.075, *p* = 0.042) and higher BMI (B* = −0.208, *p* < 0.001) were significantly associated with lower HRQoL. In the model for women, the number of chronic conditions (B* = −0.055, *p* = 0.044) and BMI (B* = −0.095, *p* < 0.001) showed significant negative associations.

**Table 3:** Linear regression for EQ-5D-5L index score, stratified by gender.

Variables	Male (*N* = 416)		Female (*N* = 613)	
	B*	*p* value	B*	*p* value
Exposure status (yes)	0.034	0.315	0.017	0.476
Age (years)	**−0.137**	**<0.001**	0.013	0.639
Number of conditions (by body system)	**−0.075**	**0.042**	**−0.055**	**0.044**
BMI	**−0.208**	**<0.001**	**−0.095**	**<0.001**
ADL independence (BI)	**0.499**	**<0.001**	**0.606**	**<0.001**
Hamilton anxiety rating scale	**−0.259**	**<0.001**	**−0.347**	**<0.001**
Change in daily routines				
No-change	Ref		Ref	
Basic-only	−0.023	0.504	−0.024	0.345
Care-only	**−0.081**	**0.021**	−0.002	0.920
Social-only	−0.066	0.068	0.000	0.998
Basic-care	**−0.101**	**0.003**	−0.041	0.100
Basic-social	−0.026	0.453	**−0.052**	**0.041**
Care-social	−0.050	0.158	−0.005	0.851
All	−0.061	0.104	**−0.055**	**0.003**

B*, Standardised beta coefficients. All models were adjusted by the 11 autonomous regions. Bold values indicate statistical significance (*p* < 0.05).

Functional independence, measured by the Barthel Index, showed the strongest positive association with HRQoL in both men (B* = 0.499, *p* < 0.001) and women (B* = 0.606, *p* < 0.001). Anxiety symptoms, assessed with the Hamilton scale, were negatively associated with HRQoL in all genders, with a slightly stronger effect in women (men: B* = −0.259, *p* < 0.001; women: B* = −0.347, *p* < 0.001).

Regarding changes in daily routines, men showed significant associations between lower HRQoL and changes in healthcare routines (B* = −0.081, *p* = 0.012) and combined basic and healthcare routines (B* = −0.101, *p* = 0.003). Among women, changes in combined basic and social routines (B* = −0.052, *p* = 0.041) as well as in all three routine types (B* = −0.076, *p* = 0.006) were significantly associated with lower HRQoL.

## Discussion

To our knowledge, this is the first study to examine the impact of the COVID-19 pandemic on daily routines among older adults and its relationship with health-related quality of life from a gender perspective. We recruited a sample of 1,072 community-dwelling individuals aged 75 and older, with a remarkably high level of functional independence (96% classified as fully independent). The prevalence of moderate or substantial changes in daily routines was considerable, particularly in social routines, and these changes did not appear to be associated with the time elapsed since the interviews. Women reported significantly more frequent changes across all routine types. However, the pattern of associations with lower HRQoL differed by gender: in men, significant associations were observed for disruptions in healthcare routines and in combined basic and healthcare routines, while in women, lower HRQoL was linked to disruptions in combined basic and social routines and when all three types of routines were affected simultaneously. These findings suggest gender-specific vulnerabilities, with men being potentially more sensitive to care-related disruptions, whereas women appear more affected by routine disruptions involving social domains or multiple areas of daily life.

When comparing the results of this study with data from the pre-pandemic Spanish National Health Survey (26) for the 75 to 85 age group, men showed a higher proportion reporting no mobility problems (65.00% vs. 60.43%), suggesting a potential improvement in this dimension. However, both men and women in our sample exhibited a worsening in the dimensions of Pain/Discomfort and Anxiety/Depression. A recent scoping review analysing seven studies on social isolation, loneliness, and health-related quality of life in older adults during the COVID-19 pandemic concluded that perceived social isolation due to restrictions had a negative impact on loneliness and HRQoL, which could explain these differences from pre-pandemic data.

Similarly, a study conducted in Taiwan analysed the impact of social situation, self-rated physical health, sleep quality and residential stability on the quality of life of older adults during the COVID-19 pandemic (27). The authors concluded that HRQoL was negatively affected by a shortage of social interactions, poor sleep quality, cohabitation with relatives with mobility problems, frequent changes in place of residence and lower income levels. Another study conducted in Brazil aimed at describing the quality of life of older adults before and during the COVID-19 pandemic, focusing on physical activity, sedentary time and sleep quality during home confinement, obtained similar results (28). Specifically, a greater burden of illness and increased time spent sitting were associated with lower HRQoL.

This heightened vulnerability among older adults, particularly in the physical dimension of quality of life, was also observed in a study conducted in Spain (29). The authors reported that individual aged over 70 experienced greater physical vulnerability during the pandemic, largely due to isolation and pre-existing health and immunity issues. These findings are consistent with our results and highlight the importance of addressing the specific physical and social challenges faced by older adults following a public health crisis.

Physical activity and social participation are essential for maintaining both physical and mental health in older adults. The 2020 WHO guidelines on physical activity and sedentary behaviour recommended minimising sedentary time and replacing it with any level of physical activity in this age group (30). The UK Chief Medical Officers’ guidelines advocate for a combination of moderate and/or vigorous aerobic activity and strength training, as well as breaking up periods of inactivity with light activity such as household chores or shopping (31).

These daily activities not only support physical functioning and reduce the risk of falls, but are also associated with increased well-being and improved mental health (32). Physical activity and social participation are closely interrelated and often depend on maintaining daily routines. Routine activities such as grocery shopping, taking out the trash, or going to the hairdresser provide opportunities to stay active and socially connected. Being an active member of the community and maintaining autonomy in daily responsibilities provides a sense of self-fulfilment and belonging, which has been linked to greater life satisfaction and better quality of life (15). Social participation has also been associated with increased muscle mass, improved cognitive function, reduced comorbidity, prevention of disability, increased social support, decreased psychological distress and ultimately improved HRQoL (10).

The COVID-19 pandemic constituted a period of unprecedented reconfiguration of daily activities in the older populations living in the community. Both government-imposed restrictions and fear of infection led to reductions in both physical activity—especially outdoors—and social interactions (33). The pandemic also constituted a major environmental stressor, affecting access to health care for chronic diseases (e.g., for medication adjustment), hindering mobility, leisure activities and social contact. This external stressor was likely to generate a misalignment between one’s capacity and the environment, promoting the development of new disabilities (9). Older individuals are particularly vulnerable to the physical decline triggered by the pandemic, which may occur rapidly after periods of inactivity (34). During home confinement in 2020, a substantial decrease in overall physical activity was observed in the adult Spanish population, particularly for walking (35). In Spanish older cohorts it was observed that the most substantial changes in healthy habits during confinement were for physical activity and sedentary time (8). Several European and U.S. studies on older adults confirmed that self-reported physical activity (walking, moderate and vigorous exercise) and time on feet decreased substantially in the first years after the epidemic onset (31, 36).

However, few studies had previously analysed changes in light physical activity or routines, which may be critical in avoiding sedentary behaviours and maintaining social connections. Fors Connolly assessed changes in three daily activities (shopping, walking, and visiting family or friends) among European adults over 50 and found that changes in shopping and walking had greater significant repercussions on mental health (anxiety and depression) than changes in visits to relatives (32). Goverover et al. examined changes in 26 daily activities and their relationship with HRQoL, finding that individuals who changed routines the least —or adapted them to pandemic conditions—reported better quality of life (15). Otto et al. studied the change in the lives of U.S. cancer survivors due to the COVID-19 pandemic and its association with HRQoL (FACTG7), and found that those whose access to healthcare was most affected and whose routines suffered the most disruption reported a lower quality of life (37). These studies, however, were conducted in the early stages of the pandemic and focused on immediate effects. In contrast, our results suggest that pandemic-related disruptions in daily lives may persist in the medium or long term as, on the one hand, changes in routines were reported after the change in the COVID-19 surveillance strategy (38) and, on the other hand, no statistical association was observed between time since the interview and the reported change.

In our sample, women reported significantly more changes in routines than men, notably in basic and healthcare activities. Olofsson et al. similarly found that women experienced greater changes than men in all assessed routines, with a much larger effect for the reduction of shopping compared to the other activities (39). Other studies assessing the determinants of physical activity changes in older adults during the pandemic also found greater reductions in exercise levels and time on their feet among women (14). This difference may be explained by a higher sense of responsibility in the context of a crisis and greater adaptability, as women are more likely than men to implement preventive measures (39). Women may have been more compliant with the restrictions due to a greater fear of the disease, which has been observed previously (40) and has been confirmed in this study.

The largest differences in our study regarding change in routines were found for basic routines and healthcare routines and, in this context, it is possible that gender roles may have played a part. Older women are typically more involved than men in caregiving and household responsibilities, largely due to long-standing gender norms and expectations. As a result, they tend to dedicate more time to domestic tasks and care work, even in households where men are present or contribute (41, 42) In this sense, as women are more frequently involved in basic and caregiving tasks than men, they may have been more likely to notice changes in day-to-day activities during the pandemic. Furthermore, societal expectations and a strong sense of obligation to fulfill these roles may have led them to modify or interrupt their routines more than men, contributing to the higher frequency of reported routine changes (43). Their responsibility for the care of others may also have made them more likely to follow pandemic-related restrictions to protect those under their care.

Our results are subject to potential reverse causality between change in routines due to the pandemic and HRQoL. Individuals with poorer pre-pandemic health or higher disability levels may have experienced greater disruption due to a greater fear of infection or unmet healthcare needs. Also, these same individuals might have had lower resilience, more fragile mental health, and weaker social support networks, potentially triggering a more rapid deterioration of their physical condition and HRQoL as a result of the pandemic crisis (44).

The study has the limitations inherent to cross-sectional designs. The main limitation was its cross-sectional nature, which precludes causal inference and makes it vulnerable to reverse causality bias. In addition, we lacked pre-pandemic data on health-related quality of life and disability, which limits comparisons. Future studies using longitudinal designs would help to establish the directionality of associations, while qualitative approaches could provide further insights into the mechanisms underlying these observed changes. The recruitment of a representative sample of older adults (aged 75 and over) during a pandemic also constituted a significant logistical challenge and was the cause of the long recruitment period.

Another important limitation of this study was the use of non-validated questions to assess the impact of the COVID-19 pandemic on daily routines. A pragmatic approach was chosen to capture, in a clear and concise manner, the perceived changes in key areas of daily life (basic self-care, health management and social relationships), which were particularly relevant for the older populations during the pandemic. Although these questions are not part of formally validated psychometric instruments, they were designed using clear and structured wording to ensure comprehension by older adults. Given their self-reported nature, these questions are suitable for enabling comparability between groups and exploring associations between changes in daily routines and HRQoL. Nonetheless, the lack of formal validation may limit the reliability and generalizability of our findings. Future research should aim to develop standardized, validated tools for assessing changes in daily routines among older adults, in order to enhance reliability and validity. Finally, although two participants identified as non-binary and were retained in overall descriptive statistics, their small number precluded meaningful stratified or regression analyses, limiting the representation of gender diversity in our study. Future research should aim to include larger and more diverse samples of older adults to better capture the experiences and health outcomes of non-binary individuals.

Nonetheless, this study also has considerable strengths. The interviews of the participants were conducted by trained nurses using questionnaires widely used in their daily practice. Moreover, these clinical nurses were the primary care providers of the participants at their health centres, which allowed them to have a solid knowledge of the individuals’ health status and personal circumstances. This likely contributed to improving the accuracy of the data collected and minimising information loss. Sampling was carried out consecutively, minimising potential selection bias.

## Conclusions

This study provides novel evidence on the impact of the COVID-19 pandemic on daily routines and health-related quality of life among community-dwelling Spanish adults aged 75 and older, with a focus on gender differences. Our results show that Spanish older adults experienced substantial and persistent changes in their daily routines, particularly in social activities, and that these changes were associated with HRQoL outcomes.

Gender-specific patterns were observed. Women reported more frequent changes across all routine types. In men, HRQoL was more sensitive to disruptions in healthcare routines and when basic and healthcare routines were simultaneously affected. Among women, lower HRQoL was observed when basic and social routines were disrupted together, as well as when all three routine types were simultaneously affected. Importantly, the association between changes and HRQoL was found to be independent of COVID-19 infection status, suggesting that the broader social and environmental consequences of the pandemic had a greater impact on perceived heath than the infection itself.

These findings underscore the importance of incorporating a gender perspective in public health responses to future crises. Strategies aimed at preserving or restoring daily routines, especially those related to healthcare and social participation, may be key to maintaining autonomy and health-related quality of life in older populations. Moreover, it is crucial to develop personalized policies and support systems that address the diverse needs of older adults. Efforts to empower them through improved digital literacy and to create strategies that prevent and manage unwanted loneliness will be essential for enhancing resilience and well-being in this population.

## Data Availability

The raw data supporting the conclusions of this article will be made available by the authors, without undue reservation.

## Group members of CUIDAMOS+75 group

Avendaño Céspedes, Almudena. Cortés Zamora, Elisa Belén. Hidalgo Alsina, Gemma. Puig Fitó, Marta. Rami Badia, Maria Rosa. Obis Aguilar, Maria Eugènia. Guiu Bueria, Anna. Palau Morales, Maria Teresa. Fabregat Julvé, Inmaculada. Bernabeu Aicart, Clara. Mestre Tena, Juan Miguel. Tomas Brea, Marta. Parreño Membrado, María Dolores. Gimeno Escuder, Carla. Moliner Monedero, Ana. Cano Pérez, Alberto. Martínez Arroyo, Anunciación. Sánchez Ramos, Carmen Luzdivina. Regacho Ramírez, Carolina. Carretero Sierra, Consuelo. Villanueva Sanz, Cristina. Verdagay Romero, David. Ruiz García, Diego. Monteagudo García, Elena. Rojas Barra, Elena. Herrero Sancho, Elvira. Luna Espejo, Esther. Díez Rodríguez, Gema. Leyva Vera, Gloria. Nuevo Pozo, Gonzalo. Melero Redondo, Ines. Moreno García-Herrera, Inmaculada. Careaga González, Isabel. Villanova Cuadra, Laura. Ríos Ruiz, Lorena. Huerta Galindo, Luis. Almodovar López, María Ángeles. Hernández de Luna, María Concepción. Adillo Montero, María Isabel. Santamaría Martín, María Jesús. Romero Molina, María Luisa. Kamal Moreno, Nadia. Garrido Calleja, Paloma. Vadillo Obesso, Pedro. López Zarza, Raquel. Razola Rincón, Raquel. López Sánchez, Rocío. Lobato Márquez, Samuel. Criado Jorge, Sara. Morcillo de la Cuadra, Silvia. Susmozas Rodríguez Barbero, Teresa. Carpio Ibáñez, Ana María. García Gómez de Cadiñanos, Elena. Company Sancho, Maria Consuelo. Mendoza Sánchez, Rita. García Delgado, María José. Monzón Monzón, Guillermo. Díaz Díaz, Maria Isabel. Suárez Hernández, María Dolores. Candela Angulo, Beatriz. Martín Andrade, Maria Paula. Jorge Araujo, Pedro. Santana Aguiar, Eduardo. Morales Montesdeoca, Sandra. Figueroa Martín, Laura. Galdona Luis, Haridian. Martínez Mata, Silvia. Mellado Tirado, Rosa. Parrilla Suárez, Irene. Ramos Santana, Sara. Plasencia Delgado, Victoria. Rodríguez Hernández, Ingrid. Padilla Maestre, Jorge Rafael. Garrido Bollo, Sergio. Martínez de la Torre, Teresa. Guzmán Castilla, Inmaculada. Artieda Cadena, María. Ayesa Urtiaga, Vanesa. Berasain Erro, Itziar. Zubillaga Beorlegui, Anamaría. Ilundain Razquin, Graciela. Gutiérrez Pardo, María Carmen. Lucea Matinez, Maite. Magno Villanueva, Mónica. Martínez Zaborras, María de Leyre. Martínez Cabodevilla, María Jesús. Galán Espinilla, María José. Ariztegui Echenique, Ana María. Mateo Cervera, Ana María. Cidoncha Moreno, Mª Ángeles. Moreno Leal, Maribel. Barrenetxea Lejarreta, Ana Isabel. Albestegi Soto, Asier. Iglesias Domínguez, María Félix. Gallo Alcalá, Ruth. Carrera Alfonso, Elisabet. Pisà Gaia, Mara. Miralles Xamena, Jerónima. Sánchez Jiménez, Milagros. Barea Mestre, M. Josep. García Salom, Elena. Garcia Duran, Nuria. Pons Burguera, Rosa María. Severi, Wiliam Andres. Góméz Bermejo, Tamara. Campos Guerrero, Ana Belén. Perelló Fuster, Marta. Hernandez Yeste, M. Soledad. Terrón Rodriguez, Elena. De la Rosa López, Laura. Cantos Garcia, Encarnación. Arjona Hernández, Fuensanta. De Castro Día, Maria Milagros. Polo Quiles, Pilar. Soriano Campos, Rita Isabel. Chumillas López, Josefa. Fernandez Abellan, Maria Jose. Valverde Jimenez, Maria Rosario. Muñoz Garcia, Maria Jose. Cerezo Sanmartin, Margarita. Morales Nicolas, Dolores. Laveda Lopez, Maria Jose.
