# Supplementary material for: Changes in daily routines and health-related quality of life among Spanish older adults after the COVID-19 pandemic: analysis from a gender perspective
Source: Front Public Health. 2025 Oct 13;13:1678003. doi: 10.3389/fpubh.2025.1678003 (PMC12554437; doi:10.3389/fpubh.2025.1678003)
Supplement: Supplementary file 2 [file Table_2.docx]

# Additional file 2- Electronic questionnaire (Translated version).

**Compliance with inclusion criteria**

Date of data collection

Cognitive impairment

(INSTRUCTION: The answer is affirmative if the medical history contains at least one nursing or medical diagnosis confirming this situation. If not, you can administer the Pfeiffer questionnaire to determine inclusion)

Yes No


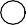

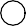


Institutionalised patient

Yes


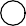

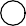


No

Barthel Index in medical history < = 5

Yes No


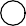

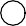


Expected survival less than 9 months

Yes No


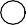

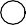


Psychiatric condition that precludes participation in the study

Yes No


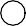

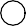


Exposed: SARS-CoV2 infected patients with confirmed positive diagnosis after 11/05/2020 and before 01/06/2022. (Positive confirmatory test for nucleic acid amplification (rRT-PCR) or antibody who have been symptomatic for < 5 days with a positive Ag test)

Unexposed: Patients not infected with SARS-CoV2 in the same time period.

Identify whether the participant belongs to the exposed or non-exposed group:


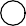
 Exposed
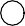
 Non-exposed

**Basic sociodemographic and clinical variables**

Date of birth

Current age

Gender Woman

Man


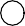

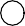

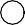


Non-binary

**Comorbidities grouped by body system**

Yes No


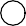

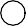
Neoplasias


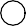

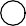
Blood and immune system diseases


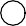

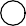

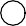

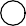
Endocrine diseases

CNS diseases


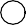

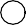


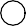

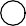
Sensory diseases


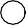

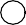
Cardio-circulatory system diseases


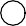

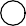
Respiratory system diseases


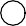

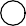
Digestive system diseases


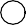

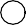
Skin diseases


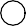

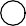
Musculoskeletal and connective tissue diseases


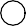

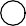
Genito-urinary system diseases

**Clinical Variables**

Estimated weight (kg)

Estimated height (cm)

BMI

**Social variables**

Home cohabitation status As a couple


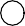

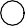

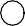


Lives with a relative

Lives with others (friends, colleagues)

Lives with a caregiver


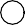

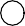


Alone

Does he/she have a caregiver? Unpaid family/friend Remunerated

Both


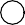

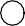

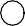

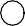


No

Has he or she lost family or friends due to the pandemic?

Yes No


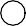

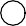


**Changes in routines**

How do you think the COVID pandemic has influenced your routines related to basic needs, such as eating, sleeping, dressing and hygiene habits?

(INSTRUCTION: Including shopping/culinary habits, number of times you go shopping, frequency of cooking, sleep schedules and quality of sleep, body image care, hygiene habits, visits to hairdresser)

No change Little change Considerable change Substantial change


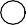

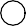

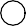

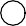


How do you think the COVID pandemic has influenced your routines related to health care and care for others?

(INSTRUCTION: This includes visits to the health centre or other health services (podiatrist, dentist, ...), individual physical activity, as well as taking full or partial care of family members or relatives (e.g. spouse or grandchildren)).

No change Little change Considerable change Substantial change


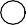

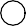

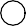

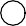


How do you think the COVID pandemic has influenced your routines related to social relations?

(INSTRUCTIONS: Including play activities such as routine meetings with peers, games, snacks/breakfasts/meals, meetings with acquaintances/friends, senior centre activities, physical activity in groups)

Not change Little change Considerable change Substantial change


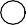

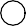

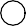

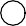


# COVID-19 threat perception (BIP-Q5)

| **COVID-19 threat perception (BIP-Q5)** |  | | |
| --- | --- | --- | --- |
| To what extent do you think COVID-19 affects or could affect your life? | 1 – Greatly affected | 5 | 10 – Not at all |

*(Place a tick on the scale above)*

| How long do you think the  COVID-19 disease lasts? | 1 – Greatly affected | 5 | 10 – Not at all |
| --- | --- | --- | --- |

*(Place a tick on the scale above)*

| To what extent do you experience discomfort from the disease? To what extent do you think the disease causes  discomfort? | 1 – Greatly affected | 5 | 10 – Not at all |
| --- | --- | --- | --- |

*(Place a tick on the scale above)*

| How worried are you about being infected with COVID-19? | 1 – Greatly affected | 5 | 10 – Not at all |
| --- | --- | --- | --- |

*(Place a tick on the scale above)*

| How much does or would COVID-19 infection affect you emotionally (e.g. does or would it make you feel angry, scared, upset or depressed)? | 1 – Greatly affected | 5 | 10 – Not at all |
| --- | --- | --- | --- |

*(Place a tick on the scale above)*

Please indicate in order of importance the three main factors that you consider to be causing the disease.

TOTAL SCORE - COVID-19 THREAT PERCEPTION (BIP-Q5)

# Duke questionnaire (social support)

**Duke questionnaire**

1. I receive visits from my friends and family Much less than I want


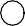

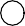

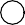

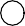

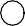


Less than I wish

Neither too much nor too little

Almost as much as I want As much as I wish

1. I receive help in matters related to my home. Much less than I want

Less than I wish


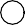

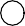

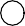

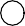

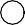


Neither too much nor too little

Almost as much as I want As much as I wish

1. I receive compliments and recognition when I do my job well. Much less than I want

Less than I wish


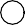

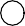

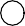

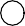

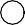


Neither too much nor too little

Almost as much as I want As much as I wish

1. I have people who care about what happens to me. Much less than I want

Less than I wish


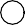

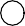

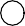

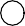

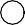


Neither too much nor too little

Almost as much as I want As much as I wish

1. I receive love and affection. Much less than I want Less than I wish

Neither too much nor too little


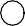

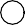

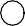

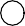

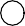


Almost as much as I want As much as I wish

1. I have the possibility to talk to someone about my Much less than I want

problems at work or at home Less than I wish


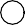

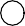

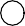

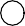

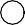


Neither too much nor too little

Almost as much as I want As much as I wish

1. I have the possibility to talk to someone about my Much less than I want

personal and family problems Less than I wish


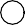

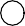

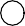

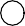

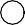


Neither too much nor too little

Almost as much as I want As much as I wish

1. I have the possibility to talk to someone about my Much less than I want financial problems Less than I wish

Neither too much nor too little


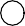

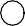

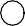


Almost as much as I want As much as I wish

1. I receive invitations to be entertained and to go out with Much less than I want other people Less than I wish

Neither too much nor too little

Almost as much as I want As much as I wish

1. I receive useful advice when an important event Much less than I want happens in my life Less than I wish

Neither too much nor too little

Almost as much as I want As much as I wish

1. I get help when I am sick in bed Much less than I want

Less than I wish Neither too much nor too little

Almost as much as I want As much as I wish

TOTAL SCORE

# Barthel Index

**Autonomy for Activities of Daily Living (Barthel Index)**

EATING

10 - Fully independent

05 - Needs help cutting meat, bread, etc.

00 - Dependent

WASHING

05 - Independent: enters and leaves the toilet alone 00 - Dependent

DRESSING

10 - Independent: able to put on and take off clothes, to button and tie shoes. 05 – Needs help

00 - Dependent

GROOMING

05 - Independent for washing face, hands, combing hair, shaving, make-up, etc. 00 - Dependent

DEPOSITIONS

(assess this over the previous week)

10 – Normal continence

05 - Occasionally an episode of incontinence, or needs help administering suppositories or enemas

00 - Incontinence

MICTION

(assess this over the previous week)

10 - Normal continence, or is able to care for the catheter if he/she has one

05 - A maximum of one episode of incontinence per day, or needs help with catheter care 00 - Incontinence

USING THE TOILET

10 - Independent to go to the toilet, take off and put on clothes... 05 - Needs help to go to the toilet, but cleans up after himself/herself

00 - Dependent

*Page 2*

TRANSFERS

15 - Independent to go from couch to bed

10 - Minimal physical assistance or supervision to do so

05 – He/she needs a lot of help, but is able to sit up on his/her own. 00 - Dependent

MOBILITY

15 - Independent, walks alone 50 metres

10 - Needs physical assistance or supervision to walk 50 metres. 05 - Independent in wheelchair without assistance

00 - Dependent

STAIRS

10 - Independent for walking up and down stairs

05 - Needs physical help or supervision to do so 00 - Dependent

TOTAL SCORE – BARTHEL INDEX

# Anxiety questionnaire (Hamilton)

**HAMILTON ANXIETY SCALE**

1. ANXIOUS MOOD 0 (No)

1

2

3

4 (Severe)

(Restlessness/Expecting the worst/Apprehension

-Irritability )

1. TENSION 0 (No)

1

2

3

4 (Severe)

(Feelings of tension/Fatigue/Impossibility to relax/Easy crying/Trembling)

1. FEARS 0 (No)

1

2

3

4 (Severe)

(of the dark/strangers/being left alone/large animals/crowds)

1. INSOMNIA 0 (No)

1

2

3

4 (Severe)

(Difficulty falling or staying asleep/Tiredness on waking/Nightmares or night terrors)

1. INTELLECTUAL FUNCTIONS 0 (No)

1

2

3

4 (Severe)

(Difficulty concentrating/Poor memory)

1. DEPRESSED MOOD 0 (No)

1

2

3

4 (Severe)

(Lack of interest/Anhedonia/Depression)

1. MUSCULAR SOMATIC SYMPTOMS 0 (No)

1

2

3

4 (Severe)

(Muscle stiffness/Clonic jerks/Teeth grinding)

1. SENSORY SOMATIC SYMPTOMS 0 (No)

1

2

3

4 (Severe)

(Tinnitus/Blurred vision/Sniffling or chills/Tingling)

1. CARDIOVASCULAR SYMPTOMS 0 (No)

1

2

3

4 (Severe) (Tachycardia/Palpitations/Chest pain/Extrasystoles)

1. RESPIRATORY SYMPTOMS 0 (No)

1

2

3

4 (Severe)

(Chest pressure/Choking or shortness of breath/Sighing)

1. GASTROINTESTINAL SYMPTOMS 0 (No)

1

2

3

4 (Severe)

(Dysphagia/Nausea or vomiting/Stomach/Constipation/Weight loss/Abdominal fullness)

1. GENITOURINARY SYMPTOMS 0 (No)

1

2

3

4 (Severe)

(Amenorrhoea or metrorrhagia/Frequent urination/Impotence or premature ejaculation/Frigidity)

1. AUTONOMIC SYMPTOMS 0 (No)

1

2

3

4 (Severe)

(Dry mouth/Redness/Hotness/Excessive sweating/Dizziness and headaches)

1. BEHAVIOUR AT THE INTERVIEW 0 (No)

1

2

3

4 (Severe)

(Nervous agitation/Trembling/Walking)

HAMILTON TOTAL SCORE (Anxiety)

# Euroqol 5D-5L

**EUROQOL 5D-5L**

Mobility I have no problem walking

I have slight problems walking I have moderate problems walking I have severe problems walking

I can’t walk

Self-care I have no problems washing or dressing myself I have slight problems washing or dressing myself

I have moderate problems washing or dressing myself I have serious problems washing or dressing myself I can't wash or dress myself

Usual activities I have no problems in carrying out my daily activities

I have slight problems in carrying out my daily activities

I have moderate problems in carrying out my daily activities

I have serious problems in carrying out my daily activities

I am unable to carry out my daily activities (Examples: working, studying, household chores, family activities or leisure time activities)

Pain/discomfort I have no pain or discomfort

I have mild pain or discomfort I have moderate pain or discomfort I have severe pain or discomfort I have extreme pain or discomfort

Anxiety/depression I am not anxious or depressed

I am mildly anxious or depressed I am moderately anxious or depressed I am very anxious or depressed

I am extremely anxious or depressed

Mark an X on the scale to indicate your state of health TODAY.

Note: 100 being the best state of health and 0 being the worst state of health.

0 50 100

*(Place a tick on the scale above)*

EQ-5D-5L score
